# Neural synchronization deficits linked to cortical hyper-excitability and auditory hypersensitivity in fragile X syndrome

**DOI:** 10.1186/s13229-017-0140-1

**Published:** 2017-06-07

**Authors:** Lauren E. Ethridge, Stormi P. White, Matthew W. Mosconi, Jun Wang, Ernest V. Pedapati, Craig A. Erickson, Matthew J. Byerly, John A. Sweeney

**Affiliations:** 1 Department of Pediatrics, Section on Developmental and Behavioral Pediatrics, University of Oklahoma Health Sciences Center, 1100 NE 13th Street, Nicholson Tower, Suite 4900, Oklahoma City, OK 73104 USA; 2 Department of Psychology, University of Oklahoma, Norman, OK USA; 3 Department of Psychiatry, University of Texas Southwestern Medical Center, Dallas, TX USA; 4 Schiefelbusch Institute for Life Span Studies and Clinical Child Psychology Program, University of Kansas, Lawrence, KS USA; 5 Kansas Center for Autism Research and Training (KCART), Kansas City, KS USA; 6 Department of Psychology, Zhejiang University of Technology, Hangzhou, Zhejiang China; 7 Division of Child and Adolescent Psychiatry, Cincinnati Children’s Hospital Medical Center, Cincinnati, OH USA; 8 Center for Mental Health Research and Recovery, Montana State University, Bozeman, MT USA; 9 Department of Psychiatry and Behavioral Neuroscience, University of Cincinnati, Cincinnati, OH USA; 10 Division of Child Neurology, Cincinnati Children’s Hospital Medical Center, Cincinnati, OH USA

**Keywords:** Fragile X syndrome, EEG, Chirp, Gamma, Sensory

## Abstract

**Background:**

Studies in the *fmr1* KO mouse demonstrate hyper-excitability and increased high-frequency neuronal activity in sensory cortex. These abnormalities may contribute to prominent and distressing sensory hypersensitivities in patients with fragile X syndrome (FXS). The current study investigated functional properties of auditory cortex using a sensory entrainment task in FXS.

**Methods:**

EEG recordings were obtained from 17 adolescents and adults with FXS and 17 age- and sex-matched healthy controls. Participants heard an auditory chirp stimulus generated using a 1000-Hz tone that was amplitude modulated by a sinusoid linearly increasing in frequency from 0–100 Hz over 2 s.

**Results:**

Single trial time-frequency analyses revealed decreased gamma band phase-locking to the chirp stimulus in FXS, which was strongly coupled with broadband increases in gamma power. Abnormalities in gamma phase-locking and power were also associated with theta-gamma amplitude-amplitude coupling during the pre-stimulus period and with parent reports of heightened sensory sensitivities and social communication deficits.

**Conclusions:**

This represents the first demonstration of neural entrainment alterations in FXS patients and suggests that fast-spiking interneurons regulating synchronous high-frequency neural activity have reduced functionality. This reduced ability to synchronize high-frequency neural activity was related to the total power of background gamma band activity. These observations extend findings from *fmr1* KO models of FXS, characterize a core pathophysiological aspect of FXS, and may provide a translational biomarker strategy for evaluating promising therapeutics.

**Electronic supplementary material:**

The online version of this article (doi:10.1186/s13229-017-0140-1) contains supplementary material, which is available to authorized users.

## Background

Fragile X syndrome (FXS) is the most common single-gene cause of autism spectrum disorder with social anxiety and auditory hypersensitivity particularly common [1–4]. Despite rapid growth in knowledge of molecular mechanisms from KO mouse studies, there are no known effective treatments for FXS. Development of translational biomarkers that quantify neocortical correlates of sensory sensitivity in FXS can facilitate drug discovery by identifying non-invasive indicators of disease pathology and track treatment response. EEG/event-related potential (ERP) studies, readily performed across species, are a promising and relatively unexplored direction for this purpose in neurodevelopmental disorders such as FXS.

Reduced local circuit inhibition has been proposed as a neural mechanism for sensory hypersensitivity and neural hyper-excitability in FXS [5–7]. Specifically, gamma band activity has been associated with bottom-up sensory processing of stimulus characteristics [8] and primarily reflects local circuit GLU/GABA interactions involving excitation onto and inhibition originating from parvalbumin positive (PV+) fast-spiking interneurons [9, 10]. In *Fmr1* KO mice, prolonged persistent gamma activity or “UP” states have been associated with decreased glutamatergic drive onto fast-spiking GABAergic inhibitory neurons in sensory cortex [5, 6]. Further, network inhibition during UP states is less synchronous, particularly in gamma frequencies. Broadened high-frequency tuning curves suggestive of increased nonspecific excitability also have been found in *Fmr1* knockout mice [7]. Prolonged asynchronous UP states suggest that the ability to synchronize gamma power may be specifically reduced, but simultaneously net gamma power may be increased because of increased nonspecific excitability in the gamma range. These findings suggest a pattern of increased total high-frequency (gamma) neural activity but reduced temporally synchronous and spatially focused neural activity that may have broad neurobehavioral implications in addition to its impact on sensory processing [11]. Since gamma is the primary working frequency range of the human auditory system, it is possible to investigate hypotheses generated by pre-clinical models in a relatively non-invasive fashion in FXS using auditory processing paradigms and EEG [12].

Our previous findings showed significantly increased nonspecific gamma activity (gamma single-trial power) in FXS that was associated with a decreased ability to transiently synchronize evoked gamma (the “gamma spike” during early stimulus registration) and to habituate the neural response to repeated tones [13]. Related findings have been reported in *Fmr1* KO mice [14]. Our findings linking gamma power and sensory hypersensitivities in FXS patients suggest a convergence with FXS animal model findings of altered local inhibitory networks in and highlight the need for more systematic study of gamma-band neural activity in FXS.

One previously unexplored strategy is to drive sensory cortex with stimuli oscillating at gamma frequencies to examine the ability to synchronize neural responses to oscillating frequencies of sensory input. When presented with an amplitude-modulated stimulus oscillating at a certain frequency, neural networks oscillate in time with the stimulus frequency, effectively increasing the signal-to-noise ratio (SNR) for activity at that frequency in the local cortical network and thus in the EEG signal. The ability to “drive” cortical networks at desired frequencies to evaluate their functional integrity is especially important when studying high frequencies such as gamma, as neural oscillatory power is lower at high frequencies [15]. Rather than studying a single frequency as in steady-state examinations, a “chirp” stimulus that increases linearly in frequency across the stimulus presentation period enables the study of neural synchronization across a broad range of input frequencies in a short amount of time.

The current study utilized a chirp stimulus to evaluate neural synchronization to sensory input across a range of frequencies from 1 to 100 Hz. Although relatively novel in neurophysiological research, chirp stimuli have been used successfully to drive and examine neural activity in the gamma frequency range in humans [16] and rodents [17], and they have been demonstrated to be sensitive to pharmacological manipulation [18]. We predicted that FXS patients would be less able to synchronize neural networks oscillations to match the chirp stimulus that such abnormalities would be most pronounced in the gamma band and that this abnormality would be related to total background gamma power and clinical reports of sensory hypersensitivities.

## Methods

### Participants

Seventeen adolescents and adults with full mutation FXS (mean age = 26.2, SD = 11.8; age range 12–57; 4 females) and 17 age- and sex-matched controls (mean age = 26.9, SD = 10.9; age range 11–55; 4 females) participated in the study (Table 1). While genetic effects of FXS are more complex in females, we included females in primary analyses and confirmed effects in supplemental analyses of male participants. Healthy controls had no known prior diagnosis or treatment for neuropsychiatric illness. Exclusion criteria included history of seizures and current use of anticonvulsant medications including benzodiazepines or novel potential treatments for FXS (e.g., minocycline). Four patients were receiving atypical antipsychotics (2 aripiprazole, 2 risperidone), two antidepressants, (paroxetine, citalopram) and one both an atypical antipsychotic (aripiprazole) and an antidepressant (citalopram), all on a stable dose for at least 4 weeks. EEG studies of these drugs in psychiatric research suggest they do not significantly alter electrophysiology in a way that would confound data interpretation, consistent with our analyses, which only found one difference in EEG measures based on whether patients were taking these psychiatric medications [19, 20].

**Table 1:** Participant characteristics

FXS *n* = 17				Controls *n* = 17				
	Mean	Std. dev.	Range		Mean	Std. dev.	Range	*t* statistic (df)
Age	26.2	11.8	12–57	Age	26.9	10.9	11–55	*t*(32) = 0.2, *p* = .86
Full scale IQ	55.6	16.3	47–94	Full scale IQ	104.8	13.7	82–123	*t*(32) = 9.5, *p* < .001
Verbal (scaled)	2.8	3.5	1–11	Verbal	107.4	11.5	90–125	
Nonverbal (scaled)	2.1	2.1	1–7	Performance	108.6	10.8	89–124	
Deviation IQ				Deviation IQ				
Verbal	−3.4	1.8	−5.5–.15	Verbal	–	–	–	
Nonverbal	−4.7	2.2	−8.2– −1.2	Performance	–	–	–	
SCQ	21.8	6.7	14–31	SCQ	4.4	4.6	1–17	*t*(22) = 7.5, *p* < .001
Sensory profile	32.1	10.1	18–46	Sensory profile	22.6	4.9	16–30	*t*(21) = 3.2, *p* = .004

IQ assessed by Stanford-Binet in FXS and estimated using the Wechsler Abbreviated Scale of Intelligence (WASI) in healthy controls. Deviation IQ is only recommended for assessing floored subscale score IQ values for the Stanford-Binet based on population statistics provided for public use by the publisher; therefore, deviation IQ measures are not available for the healthy controls. *SCQ* Social and Communication Questionnaire

The Adolescent and Adult Sensory Profile [21] and the Social and Communication Questionnaire (SCQ; [22]) were completed for FXS participants by their primary caregiver. IQ of FXS participants was assessed with the Stanford-Binet Intelligence Scale 5^th^ Ed. [23] which has psychometric properties that enable characterization of intellectual ability in lower-IQ individuals [24]. IQ of controls was estimated using the briefer Wechsler Abbreviated Scale of Intelligence [25]. Most FXS patients scored in the intellectually disabled range, with four female patients scoring in the low-normal range; EEG and clinical ratings did not differ for these patients, and they were retained in primary analyses as planned. Due to the preponderance of low IQ scores in the FXS patients, deviation scores were also calculated for verbal and nonverbal IQ using the technique proposed by Sansone and colleagues [24]. All participants provided written informed consent (caregiver with assent or individual consent as appropriate) prior to participation, as approved by the UT Southwestern Institutional Review Board.

### Procedure

The auditory chirp stimulus used in the present study consisted of a 1000-Hz carrier tone amplitude modulated by a sinusoid linearly increasing in frequency from 0–100 Hz over 2000 ms [16]. These were presented 200 times each separated by an inter-trial interval randomly jittered between 1500 and 2000 ms. Stimuli were delivered at 65 db SPL through headphones while participants underwent EEG. Participants watched a silent movie to facilitate compliance as in prior studies [13, 26].

### ERP recording

EEG was continuously recorded and digitized at 512 Hz, with a 5th-order Bessel anti-aliasing filter at 200 Hz, using a 128 channel BioSemi ActiveTwo system (BioSemi, Inc., Amsterdam, Netherlands) with sensors placed according to the International 10/10 system [27]. All sensors were referenced to a monopolar reference feedback loop connecting a driven passive sensor and a common-mode-sense active sensor, both located on posterior scalp.

### EEG analysis

Raw data were visually inspected offline. Bad sensors were interpolated (no more than 5% per subject) using spherical spline interpolation implemented in BESA 6.0 (MEGIS Software, Grafelfing, Germany). Data were digitally filtered from .5–120 Hz (12 and 24 db/octave rolloff, respectively; zero-phase; 60 Hz notch) and re-referenced to average reference. Eye movement, cardiac, and muscle movement artifacts were removed blind to participant group using independent components analysis (ICA; Infomax) implemented in EEGLAB 11 [28] in Matlab (The Mathworks, Natick, MA). Data were epoched into 3500 ms trials (−500 to 3000 ms), averaged across trials and baseline-corrected using the 500-ms pre-stimulus period. Any trial with post-ICA amplitude exceeding 100 μV was considered residual artifact and removed prior to averaging. Number of valid trials did not differ between groups (FXS M = 166.5, SD = 29.1; Control M = 179.1, SD = 17.9, *t*(32) = 1.5, *p* = .14).

**Fig. 1:**
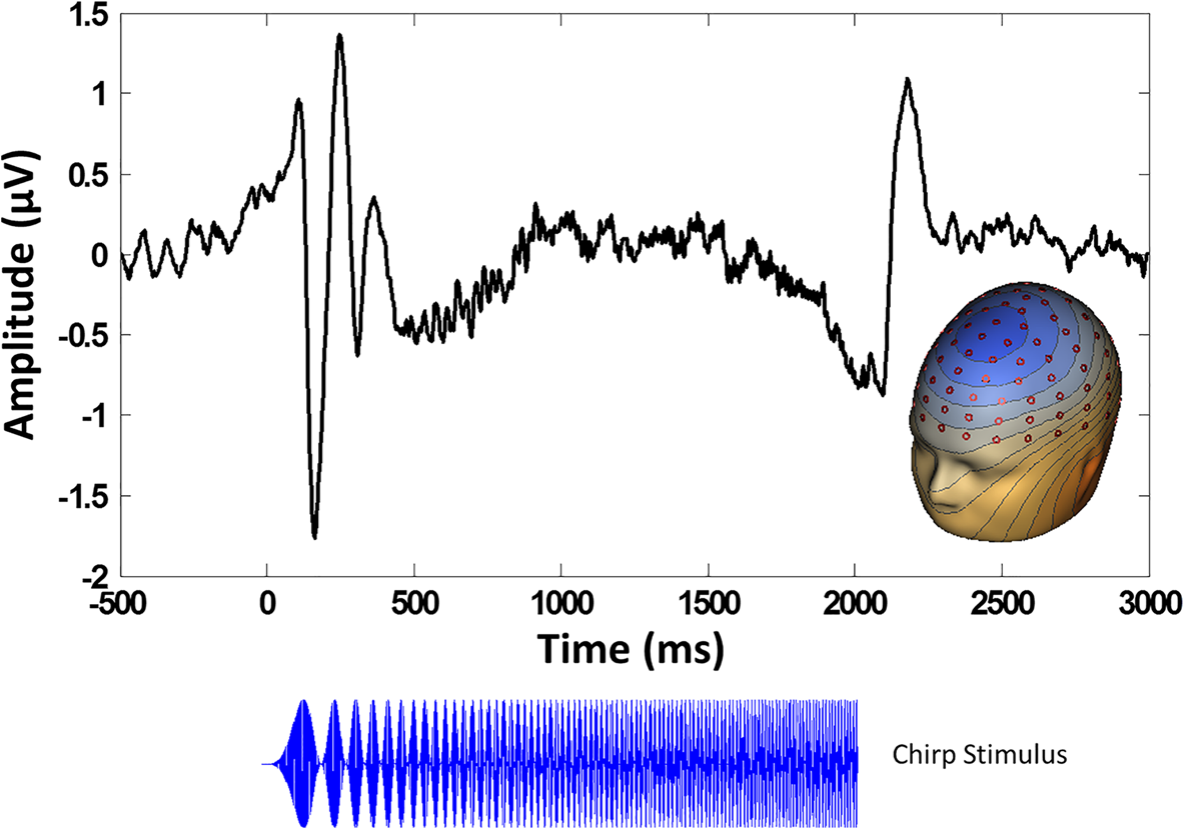
Example averaged virtual channel created by weighting the grand average epoched EEG data by the spatial PCA component, with inset PCA spatial component topography. Chirp stimulus amplitude modulation and timing is presented below. Note the increase in oscillation frequency in the PCA component waveform that matches the increase in chirp modulation frequency in the stimulus below

To take advantage of the dense electrode array and integrate data from every sensor, spatial principal components analysis (PCA) was implemented on the grand average ERP [29, 30]. PCA solutions were correlated *r* = .99 between groups, so the grand average ERP solution collapsed across group was retained. Component weights were multiplied by each subject’s average data, summed across sensors, and divided by the sum of the component weights, reducing waveforms from one for each sensor to one waveform per component with a defined spatial distribution across the scalp (Fig. 1). Spatial PCA revealed a single spatial component representing 85.3% of the variance that was consistent with known topographies representing signal from auditory cortex (Fig. 1). Subsequent analyses were performed on a virtual sensor created by weighting trial-wise EEG data by the PCA component weights.

PCA-weighted un-baseline-corrected epoched single-trial data were analyzed in the time-frequency domain using Morlet wavelets with 1-Hz frequency steps using a linearly increasing cycle length from 1 cycle at the lowest frequency (2 Hz) to 30 cycles at the highest (120 Hz). Single-trial power (STP) and inter-trial coherence (ITC) measures obtained from this method evaluated the amplitude of response at each frequency and how stable or phase-locked responses were to the auditory stimuli across trials, respectively [27]. Raw ITC values were initially corrected for trial number by subtracting the critical *r* value [1/sqrt (number of trials)] for each subject based on trial count. STP and ITC values were averaged over trials for each individual and transformed into time-frequency plots down-sampled to 250 time-bins.

### Statistical analysis

**Fig. 2:**
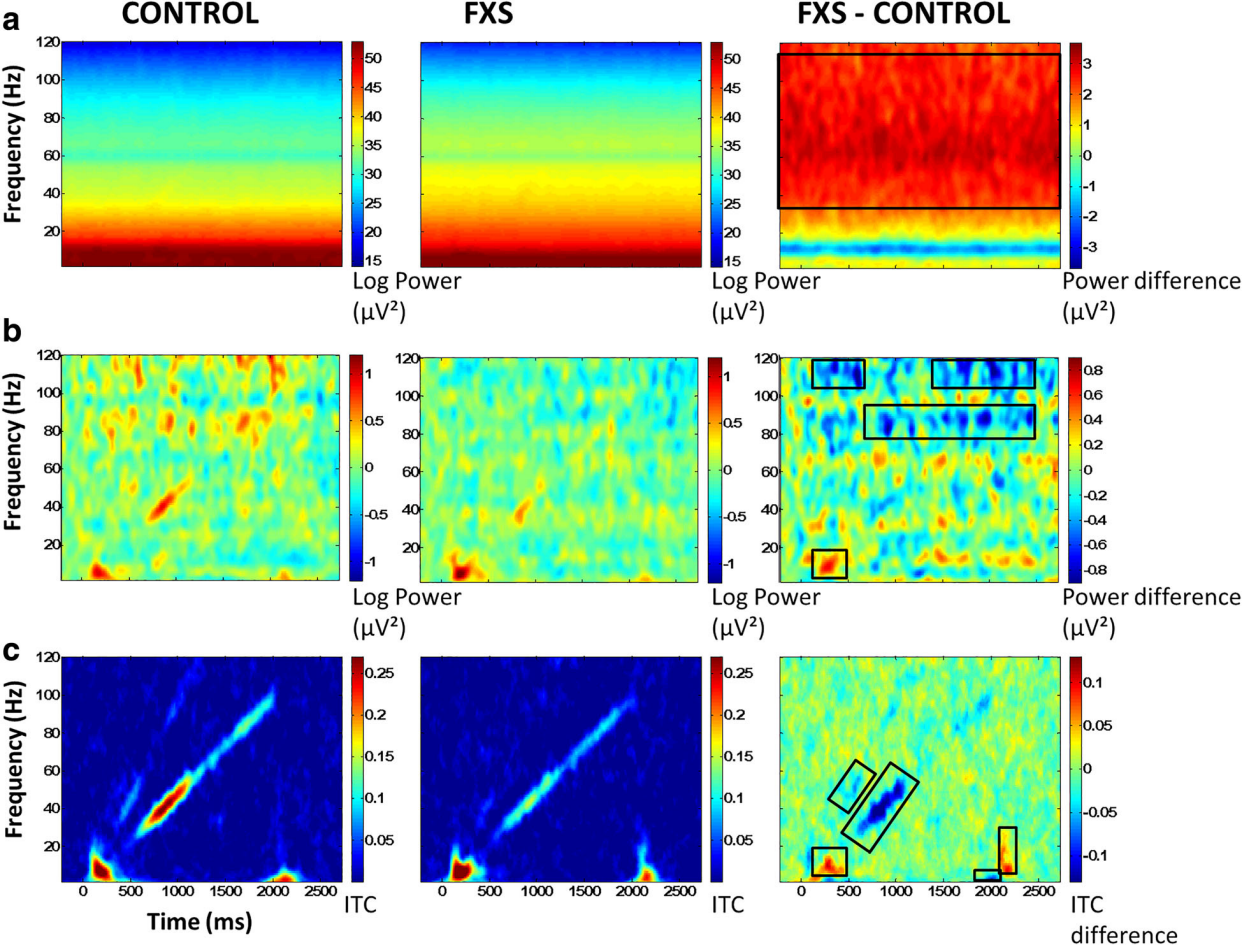
**a** Single trial power (STP) for controls, FXS, and difference maps (FXS minus controls). **b** Baseline corrected single trial power (STP) for controls, FXS, and difference maps (FXS minus controls). **c** Inter-trial coherence (ITC) for controls, FXS, and difference maps (FXS minus controls). *Black boxes* in the difference maps indicate clusters with significant group differences. Warmer colors (*reds*, *yellows*) in the difference maps (*right column*) indicate higher phase-locking or higher power for FXS, and cooler colors (*blues*, *greens*) indicate higher values for healthy controls

For stimulus-related EEG analyses, point-by-point two-tailed *t* tests were used to calculate group differences across the time-frequency matrix. Time-frequency clustering techniques and Monte Carlo simulations controlled for multiple comparisons [29, 31]. To maintain a family-wise alpha of *p* < .01 (corrected for multiple comparisons), a minimum of three sequential time-bins and three adjacent frequencies were required to be significant at a nominal threshold of *p* < .05. STP was analyzed both without baseline correction (Fig. 2a) and secondarily with baseline correction applied for each frequency (Fig. 2b).

For pre-stimulus analyses, the same component weights were applied to the pre-stimulus period in order to analyze pre-stimulus activity from the same PCA-defined topographical area in which stimulus-related activity occurred. Theta and alpha band modulation of gamma activity were of particular interest, as previous studies have suggested theta and alpha abnormalities in FXS patients [32, 33] and KO rodent models [34]. Frequency bands of interest were chosen as follows based on standard low-frequency and gamma band cut-offs: theta, 4–7 Hz; alpha, 8–12 Hz; gamma, 30–80 Hz. Additional analyses on delta and beta frequency bands are presented in Additional file 1. Coupling of low-frequency oscillations (theta, alpha) with higher-frequency oscillations (gamma) in the pre-stimulus period were evaluated to assess top-down modulation of gamma activity in local cortical networks during preparation for stimulus processing [35–37]. For amplitude-amplitude coupling analyses, for each participant, amplitude of baseline single-trial pre-stimulus data (from −1000 to −50 ms pre-stimulus, padded to avoid windowing effects) was calculated as the average amplitude (absolute value of the complex wavelet result) within a trial and within each frequency band of interest. Average amplitude was then correlated across trials for each low-frequency amplitude measure of interest (theta, alpha) with the amplitude of high-frequency power (gamma). Correlation coefficients were *z*-transformed across all participants and then compared between groups.

For phase-amplitude coupling analyses, instantaneous phase and amplitude in each frequency band were calculated from the Hilbert transformed data using the Phase-Amplitude Coupling Toolbox (PACT) for EEGLAB [38]. Trial data was concatenated for each individual to calculate overall pre-stimulus phase-amplitude coupling. Modulation index [39], a measure of cross-frequency phase-amplitude coupling, and mean resultant vector length, a measure of phase consistency in low frequencies across windows of time with the highest amplitude of gamma, were calculated according to standard methods within the toolbox (36 phase bins, top 2% of gamma amplitudes analyzed; [38]). Modulation index is sensitive to amplitude, which may differ between groups [13], whereas mean resultant vector length is normalized to [0 1]; therefore, mean resultant vector length was used for group comparisons. To be comprehensive and facilitate comparison with prior work, both measures were included in clinical correlation analyses.

Clinical correlations were examined with all significant time-frequency clusters. We also examined hypothesis-driven associations between gamma STP and gamma ITC. To examine whether pre-stimulus activity was associated with evoked response parameters of interest, pre-stimulus amplitude-amplitude and amplitude-phase coupling measures were correlated with gamma STP and gamma ITC during tone processing. All correlations were conducted using Spearman’s rho. Clinical correlations with sensory and social abnormalities (Sensory Profile, SCQ), age, and deviation IQ are presented as exploratory and hypothesis generating and not corrected for multiple comparisons.

## Results

### EEG

Point-by-point *t* tests on time-frequency plots for ITC and STP (corrected for multiple comparisons) revealed 10 time-frequency clusters with significant differences between FXS and controls (Fig. 2, Table 2). Cluster names are identified by timing relative to stimulus onset and frequency band, which provides equivalency with the *X* and *Y* axes in Fig. 2. Cluster peaks were identified using the highest *t* values reflecting group differences, not peaks of activity. For absolute gamma STP, a peak statistic is reported; however, group differences in gamma power were remarkably stable throughout the trial including before, during, and after stimulus presentation. FXS patients showed significantly increased phase-locking in alpha frequencies during the stimulus onset time period and decreased theta frequency phase-locking and increased alpha frequency phase-locking after stimulus offset (Fig. 2c), consistent with increased ERP amplitudes found in previous work [40, 41]. As expected, FXS showed decreased gamma phase-locking to the chirp stimulus from 30–58 Hz and decreased gamma phase-locking to the chirp harmonic from 47–58 Hz (Fig. 2a) as well as a decreased ability to increase gamma power above the already elevated baseline (Fig. 2b).

**Table 2:** Time-frequency clusters with significant group differences

Cluster label	Time range	Peak time	Peak frequency	Statistic	Direction of group difference
Phase-locking (ITC)					
Stimulus onset alpha	252 to 430 ms	288 ms	13 Hz	*t*(32) = 3.4, *p* = .002	FXS > CON
Stimulus offset theta	1883 to 2049 ms	1966 ms	3 Hz	*t*(32) = 3.62, *p* = .001	CON > FXS
Stimulus offset alpha	2084 to 2214 ms	2178 ms	9 Hz	*t*(32) = 3.20, *p* = .003	FXS > CON
Chirp	607 to 1068 ms	761 ms	42 Hz	*t*(32) = 2.8, *p* = .008	CON > FXS
Chirp harmonic	382 to 631 ms	406 ms	50 Hz	*t*(32) = 2.9, *p* = .007	CON > FXS
Single trial power					
Overall gamma	−220 to 2722 ms	819 ms	57 Hz	*t*(32) = 2.8, *p* = .008	FXS > CON
Baseline corrected single trial power					
Stimulus onset alpha	229 to 335 ms	276 ms	11 Hz	*t*(32) = 3.0, *p* = .005	FXS > CON
80 Hz	855 to 2462 ms	914 ms	82 Hz	*t*(32) = 3.0, *p* = .005	CON > FXS
110 Hz stimulus onset	300 to 619 ms	572 ms	112 Hz	*t*(32) = 3.0, *p* = .005	CON > FXS
110 Hz late	1647 to 2462 ms	2261 ms	112 Hz	*t*(32) = 3.1, *p* = .005	CON > FXS

Cluster names are identified by stimulus association and frequency band. Time ranges are given relative to initial stimulus onset, to provide equivalency with the *X* axes in Fig. 2. Cluster peaks are identified for highest *t* values, not for peaks of activity. For overall gamma single trial power, a peak statistic is reported; however, it should be noted that group differences were remarkably stable throughout the time period

Cross-frequency amplitude-amplitude and phase-amplitude coupling analyses during the pre-stimulus period when participants were awaiting the next chirp stimulus revealed that control subjects showed a positive cross-frequency amplitude-amplitude coupling between all low frequencies and gamma. FXS showed significantly less amplitude-amplitude coupling between alpha and gamma, *t*(32) = 2.5, *p* = .02, compared to controls. There were no group differences in theta-gamma amplitude-amplitude coupling or in the phase-amplitude coupling measures. Coupling measures were analyzed for low (30–50 Hz) and high (65–80 Hz) gamma separately, but results did not differ so this band was collapsed for final analyses.

### Gamma power and phase-locking

Although there were no group differences in number of artifact-free trials retained, gamma STP and ITC were negatively correlated with valid trial number in both controls and FXS. Therefore, these variables were corrected for trial number by subtracting the product of the linear regression coefficient for controls and trial number [42]. Trial-adjusted correlations remained significant; rho values reflect the adjusted values. For FXS, increased gamma single trial power was correlated with decreased gamma phase-locking to the chirp stimulus (rho = −.83, *p* < .001) and to its harmonic (rho = −.78, *p* < .001). Increased gamma power in FXS can be characterized as an increase in background neural “noise,” reducing overall signal-to-noise ratio (SNR) of sensory processing in the auditory cortex. This association between increased nonspecific high-frequency neural activity and decreased ability to synchronize gamma frequency activity is consistent with our previous reports from an auditory habituation task and during the resting state, but made more apparent by its relation to the gamma neural entrainment alterations during the chirp task.

Increased pre-stimulus theta-gamma amplitude coupling was correlated with increased gamma STP (rho = .55, *p* = .02) and decreased gamma phase-locking to the chirp (rho = −.69, *p* = .002) and the chirp harmonic (rho = −.63, *p* = .007).

### Clinical correlations

Significant correlations with clinical measures in FXS participants are presented in Table 3. Of particular interest, increased gamma power and reduced phase-locking to the chirp were significantly related to clinical ratings of both sensory hypersensitivities and autism spectrum social/behavioral features, respectively. Gamma STP was higher in medicated patients, *t*(15) = 2.8, *p* = .01. It is not possible to determine whether psychiatric medications were causing EEG alterations or if the medications were prescribed to treat particularly severe behavioral problems associated with the EEG alterations (medicated patients also had more abnormal clinical ratings on the Sensory Profile and SCQ—*p* < .01). However, in studies of multiple psychiatric disorders, gamma power remains relatively unaffected in patients treated with these medications [19, 20]. FXS patients showed a significant correlation between age and gamma ITC during the ERP to stimulus onset (rho = .59, *p* = .01) and age and theta ITC during the ERP to stimulus offset (rho = .52, *p* = .03), while these correlations were not significant in the age-matched controls. Controls showed significant age correlations for theta-gamma phase amplitude coupling (modulation index: rho = −.61, *p* = .009; vector length: rho = −.53, *p* = .03) that were not reflected in FXS.

**Table 3:** Significant clinical correlations with EEG findings for FXS patients

EEG measure	Clinical scale		
	Sensory profile—auditory	SCQ total score	Deviation score: nonverbal IQ
ITC chirp	NS	−.74*	NS
STP gamma	.61*	.84**	NS
Pre-stimulus theta/gamma amp-amp coupling	NS	.74*	NS
Pre-stimulus theta/gamma phase amplitude coupling—vector length	NS	NS	.58*

EEG measures with no significant correlations to clinical variables are not included. All correlations are Spearman’s rho. All correlations represent the FXS group only. *NS* Not significant

*p < .05

**p < .01

## Discussion

The current study utilized a neural entrainment approach for the first time in FXS to selectively drive networks oscillators in auditory cortex to clarify the extent of hypothesized deficits in local circuit inhibition in FXS. FXS patients demonstrated a marked reduction in the ability to synchronize (phase-lock) high-frequency neural activity to the chirp stimulus as well as the first harmonic (doubling of chirp frequency) suggesting impairments in the synchronization of networks at the primary and secondary levels of sensory processing. Both phase-locking abnormalities were highly associated with increased nonspecific gamma power, providing new evidence for a robust functional link between increased local network excitation reflected in raw gamma power and decreased ability to synchronize high-frequency population-level neural activity in sensory networks. The combination of deficits in entrainment coupled with hyper-excitation was specific to gamma, which coincide with highly selective deficits in neural synchronization related to local network excitation/inhibition balance previously identified in FXS translational models and proposed to underlie core clinical deficits in FXS [5, 43].

Increased desynchronous high-frequency firing or “noise” (gamma STP) and decreased synchronized gamma activity to a driving stimulus indicate that both factors contribute to an overall worsening of auditory cortex SNR during sensory processing in FXS. FXS patients were also unable to selectively increase gamma power above baseline compared to controls, possibly indicating that ongoing gamma power is saturated. Decreased SNR reflecting heightened neocortical excitability suggests a plausible mechanism for the highly prevalent sensory hypersensitivity found in individuals with FXS. Indeed, increased gamma STP was correlated with parental reports of increased auditory hypersensitivity. Increased gamma STP was also associated with autism-associated social impairment using the SCQ, suggesting that cortical hyper-excitability may have broader clinical impact on behavior rather than selectively impacting sensory systems. We speculate that coherent high-frequency inhibitory network interactions play a crucial role in defining receptive fields that are important for sensory, perceptual, and cognitive processes.

Recent work in *Fmr1* KO rodent models have shown a shift toward lengthened activation states and decreased synchronization of fast-spiking interneuron-to-excitatory cell networks in visual cortex [44] and reduced cross-frequency gamma coupling in hippocampus [34], suggesting that fast-spiking interneuron alterations findings may not be restricted to a specific sensory modality but may represent a more widespread neocortical abnormality. Because of the potential for an alteration in bottom-up information processing to cause a wide range of neurobehavioral symptoms, high-frequency neural activity may also represent an important treatment target for alleviating multiple behavioral and sensory abnormalities in FXS.

To investigate whether ongoing network oscillatory properties contributed to decreased sensory system SNR in FXS, we examined amplitude-amplitude and phase-amplitude coupling between low frequencies and gamma activity during the pre-stimulus period before chirp presentation. Similar to findings in *Fmr1* KO mouse cortex [34], we found a shift toward reduced influence of alpha frequency oscillations on gamma power and synchronization in FXS compared to controls. Cortical networks in healthy controls generally utilize alpha oscillations to control and inhibit local network excitation [45]; in FXS, these networks may rely more consistently on slower theta waves or even delta waves (see Additional file 1) to modulate gamma frequency, given the lack of deficit in theta-gamma coupling. This strategy may have some success, given the moderate correlation between increased theta-gamma phase-amplitude coupling and higher nonverbal deviation IQ (Table 3) and the moderate correlation between delta-gamma amplitude-amplitude coupling and more normalized gamma power and phase-locking (Additional file 1), but it may be a less successful alternative and one with potential adverse sequelae on higher order neurobehavioral processes dependent on phasic delta/theta modulation, including processes associated with ASD-like behaviors (see Additional file 1: Table S2). This shift may also account for increases in theta power and connectivity seen in resting EEG in FXS [32, 33, 46], increased gamma abnormalities during the chirp and increased pre-stimulus theta-gamma coupling.

We also identified increases in alpha frequency phase-locking during stimulus onset and offset and a decrease in theta frequency phase-locking synchronous with stimulus offset in FXS compared to controls. The increased alpha phase-locking is consistent with previous findings of increased amplitude early ERP components in response to brief auditory stimuli in FXS [26, 40, 41]. Decreased theta frequency phase-locking simultaneous to stimulus offset is a novel finding in FXS. Given the role of theta-gamma coupling in network inhibition, this theta burst at stimulus offset for healthy controls may represent activation to prepare for the inhibition required to stop the gamma oscillations when the chirp stimulus stops, a mechanism which may be deficient in FXS.

Preclinical work in *Fmr1* knockout mice has related increased gamma excitability to decreased excitatory drive on fast-spiking inhibitory interneurons, resulting in increased and poorly synchronized pyramidal cell firing in the gamma range [5]. Decreased activation of fast-spiking inhibitory interneurons, which synchronize gamma activity via projections from inhibitory neurons onto multiple pyramidal neurons, has been proposed as a mechanism for heightened neocortical excitability in FXS [5]. Poorly organized inhibitory drive onto pyramidal cells in auditory cortex from fast-spiking interneurons that control gamma synchronization could account for the pattern we observed of increased gamma power, suggesting increased high-frequency firing of excitatory pyramidal neurons (noise) but with less coherent organization (phase-locking) in response to sensory stimulation. This pattern of alterations may contribute to previously reported decreases in transient gamma phase-locking [13]. Given the similar network dynamics observed in *Fmr1* rodent models [5, 7, 14, 44], findings reported here may not only extend mouse model concepts to FXS patients, but suggest that neurophysiological measures may be useful for tracking this local circuit deficit in both mouse models and patients to foster direct translational drug development for this neurodevelopmental disorder.

Certain study limitations should be considered. First, comparative work is necessary to determine the degree to which our findings occur in other neurodevelopmental disabilities, several of which have associated sensory sensitivities as seen in FXS [47]. While evidence exists for impaired phase-locking ability, increased power, and alpha-gamma phase-amplitude coupling abnormalities in other disorders such as ASD [48–50], these studies primarily describe broad-band power abnormalities and phase-locking deficits, whereas the most salient abnormalities in FXS were seen in the gamma band (but see [51] for gamma deficits in very young boys with ASD). Berman et al. [52] also report increased alpha-gamma phase-amplitude coupling in children with ASD, an effect not replicated in FXS, although whether these differences are due to the clinical population or the age of the participants is unknown. Intellectual disability (ID) associated with FXS may also play a role in group differences, suggesting a role for these findings in other disorders associated with ID. A pattern of low-frequency power enhancement at rest has been primarily reported in ASD only for those with ID [52]. A shift from alpha to theta band activity has been reported in Down’s syndrome [53]; however, increased theta activity in children with ADHD is robust in low- and high-IQ individuals [54]. Decreased or normal gamma power in individuals with ID is more commonly reported than the increased power seen in FXS [55, 56]. Comparative studies between FXS and other forms of ID will determine the specificity of the high-frequency disruptions reported here and their relation to low-frequency function. Second, FXS participants were taking various psychiatric medications, and their potential impact on the data cannot be ruled out. However, excluding medicated patients necessarily excludes a subsample with more severe behavioral problems limiting study representativeness, and the medications are well studied in psychiatric populations without known effects as we observed in our patients. Third, our failure to detect gender differences should be considered in the context of the limited number of FXS female participants. Analyses of the male participants were largely consistent with the findings in the full sample, but future studies with larger samples of female participants should clarify gender differences in patients with FXS. Lastly, future research is needed to investigate the sensory and perceptual consequences of the identified neurophysiological alterations.

## Conclusions

Despite extensive progress in understanding the genetic alteration resulting in FXS and its biochemical and local circuit functional consequences, far more modest advances have occurred in disease understanding at the systems neuroscience level in affected individuals [57]. Further, no treatments have demonstrated clinical efficacy for FXS, and translation from rodent models to human pharmacology has been constrained by a lack of translational biomarkers to evaluate, predict, and understand drug response. The current study provides novel evidence that alterations in synchronous activity in fast-spiking inhibitory interneurons, widely reported in translational models of FXS, may contribute to important neurophysiological alterations which are measurable at the systems level in humans with FXS and are of clinical relevance. Our previous data showed decreased gamma network inhibition in evoked responses during habituation to repeated stimuli [13] and increased resting gamma power [33]. Our current report indicates altered gamma-band neural entrainment to oscillatory chirp stimuli which parallel preclinical findings in *Fmr1* KO rodent slice preparation [8] and evoked electrophysiology research [14, 44]. Our results indicate that abnormalities in sensory neurophysiology not only are clinically relevant but may be useful for in vivo studies of FXS patients to track alterations in fast-spiking cell populations known to be altered in KO mouse models as a biomarker for predicting and evaluating response to novel therapies. Although additional investigations of test-retest reliability, task effects such as stimulus and ISI length, age effects, and sex effects are warranted, gamma deficits may serve as biologically grounded and clinically relevant outcome measures in future clinical trials for FXS.

## Additional file

Additional file 1: Additional analyses for males only and for delta and beta frequency band coupling. (DOCX 15 kb)

## Abbreviations

EEG: Electroencephalography
ERP: Event-related potential
FXS: Fragile X syndrome
GLU: Glutamate
Hz: Hertz
ICA: Independent components analysis
IQ: Intelligence quotient
ITC: Inter-trial coherence
KO: Knockout
PCA: Principal components analysis
PV+: Parvalbumin positive
SCQ: Social Communication Questionnaire
SNR: Signal-to-noise ratio
STP: Single trial power
