# Erratum to: Neural synchronization deficits linked to cortical hyper-excitability and auditory hypersensitivity in fragile X syndrome

**DOI:** 10.1186/s13229-017-0150-z

**Published:** 2017-07-26

**Authors:** Lauren E. Ethridge, Stormi P. White, Matthew W. Mosconi, Jun Wang, Ernest V. Pedapati, Craig A. Erickson, Matthew J. Byerly, John A. Sweeney

**Affiliations:** 1 Department of Pediatrics, Section on Developmental and Behavioral Pediatrics, University of Oklahoma Health Sciences Center, 1100 NE 13th Street, Nicholson Tower, Suite 4900, Oklahoma City, OK 73104 USA; 2 Department of Psychology, University of Oklahoma, Norman, OK USA; 3 Department of Psychiatry, University of Texas Southwestern Medical Center, Dallas, TX USA; 4 Schiefelbusch Institute for Life Span Studies and Clinical Child Psychology Program, University of Kansas, Lawrence, KS USA; 5 Kansas Center for Autism Research and Training (KCART), Kansas City, KS USA; 6 Department of Psychology, Zhejiang University of Technology, Hangzhou, Zhejiang China; 7 Division of Child and Adolescent Psychiatry, Cincinnati Children’s Hospital Medical Center, Cincinnati, OH USA; 8 Center for Mental Health Research and Recovery, Montana State University, Bozeman, MT USA; 9 Department of Psychiatry and Behavioral Neuroscience, University of Cincinnati, Cincinnati, OH USA; 10 Division of Child Neurology, Cincinnati Children’s Hospital Medical Center, Cincinnati, OH USA

## Erratum

“It was noticed that there was an error in the reported formula for critical r value, upon publication of the original article [1]. The formula for critical r value reported as [1/sqrt(number of trials)] should be reported as sqrt[−1/(number of trials)*log(.5)].”
