# Supplementary material for: Neural synchronization deficits linked to cortical hyper-excitability and auditory hypersensitivity in fragile X syndrome
Source: Mol Autism. 2017 Jun 7;8:22. doi: 10.1186/s13229-017-0140-1 (PMC5463459; doi:10.1186/s13229-017-0140-1)
Supplement: Additional file 1: — Additional analyses for males only and for delta and beta frequency band coupling. (DOCX 15 kb) [file 13229_2017_140_MOESM1_ESM.docx]

Additional file 1

| **Cluster Label** | **Time Range** | **Peak Time** | **Peak Frequency** | **Statistic** | **Direction of Group Difference** |
| --- | --- | --- | --- | --- | --- |
| **Phase-locking (ITC)** | | | | | |
| Stimulus onset alpha | 252 to 430 ms | 288 ms | 13 Hz | t(24)=4.1, p<.001 | FXS> CON |
| Stimulus offset theta | 1883 to 2049 ms | 1966 ms | 3 Hz | t(24)=3.8, p=.001 | CON>FXS |
| Stimulus offset alpha | 2084 to 2214 ms | 2178 ms | 9 Hz | t(24)=2.4, p=.03 | FXS>CON |
| Chirp | 607 to 1068 ms | 761 ms | 42 Hz | t(24)=2.5, p=.019 | CON>FXS |
| Chirp harmonic | 382 to 631 ms | 406 ms | 50 Hz | t(24)=2.9, p=.009 | CON>FXS |
| **Single Trial Power** | | | | | |
| Overall gamma | -220 to 2722 ms | 819 ms | 57 Hz | t(24)=2.5, p=.019 | FXS>CON |

Table S1

Group analyses performed on males only for the same clusters identified in Table 2 reveal the same group differences as in the full sample (females included).

Correlations performed on males only recapitulated the same results found in the full sample: for FXS, increased gamma single trial power was correlated with a decrease in phase-locking to the chirp stimulus (rho = -.85, p<.001) and to its harmonic (rho=-.78, p=.002).

Increased theta-gamma amplitude coupling showed a similar correlation coefficient to that found in the full sample for increased gamma STP, however with fewer subjects the decreased power led to a marginal p value (rho = .52, p=.07). Correlations with increased theta-gamma amplitude coupling and decreased gamma phase locking to the chirp (rho = -.58, p=.04) and the chirp harmonic (rho=-.61, p=.03) remained significant. Clinical correlations also remained consistent: for FXS increased SCQ score was correlated with decreased ITC at to the chirp around 40 Hz (rho=-.74, p=.04), increased gamma STP (rho=.83, p=.009), and increased theta-gamma amplitude coupling (rho=.74, p=.04). Increased sensory profile scores were correlated with increased gamma STP (rho=.65, p=.02).

Additional analyses for delta and beta frequency bands.

Although modulation of gamma by the most common modulatory frequency bands, theta and alpha, were the primary variables of interest, we also computed amplitude-amplitude coupling between delta-beta, delta-gamma, theta-beta, low alpha-beta, and high alpha-beta. These analyses are meant as exploratory information for the interested reader and are not corrected for multiple comparisons. FXS showed significantly less amplitude-amplitude coupling between theta and beta, t(32)=2.1, p=.04, low alpha and beta, t(32)=3.7, p=.001, and between high alpha and beta, t(32)=4.9, p<.001, compared to controls. There were no group differences in delta-beta or delta-gamma amplitude-amplitude coupling or in the phase-amplitude coupling measures. Only delta-gamma amplitude-amplitude coupling correlated with any of the gamma power or phase locking measures for FXS, with increased delta-gamma amplitude-amplitude coupling associated with a decrease in gamma STP/noise, rho = -.58, p=.01, an increase in gamma phase locking to the chirp, rho = .50, p=.04, and an increase in gamma phase locking to the chirp harmonic, rho= .49, p=.04.

Table S2. Significant Clinical Correlations

| **EEG Measure** | **Clinical Scale** | | |
| --- | --- | --- | --- |
|  | Sensory Profile - Auditory | SCQ total score | Deviation Score: Verbal IQ |
| Pre-stimulus delta/beta amp-amp coupling | -- | .92** | -- |
| Pre-stimulus theta/beta amp-amp coupling | -- | .73* | -- |
| Pre-stimulus delta/gamma phase-amplitude coupling (Modulation Index) | -- | -- | -.50* |

Note: EEG measures with no significant correlations to clinical variables are not included. All correlations are Spearman’s rho.

-- N.S.

*p<.05

**p<.01
